# Solvent Free Synthesis of PdZn/TiO_2_ Catalysts for the Hydrogenation of CO_2_ to Methanol

**DOI:** 10.1007/s11244-018-0885-6

**Published:** 2018-01-12

**Authors:** Hasliza Bahruji, Jonathan Ruiz Esquius, Michael Bowker, Graham Hutchings, Robert D. Armstrong, Wilm Jones

**Affiliations:** 1 School of Chemistry, Cardiff Catalysis Institute, Cardiff University, Main Building, Park Place, Cardiff, CF10 3AT UK; 2 The UK Catalysis Hub, Research Complex at Harwell, Harwell, Oxon, OX11 0FA UK

**Keywords:** CO_2_ hydrogenation, Methanol, PdZn alloy, Green methanol, Hydrogen storage

## Abstract

Catalytic upgrading of CO_2_ to value-added chemicals is an important challenge within the chemical sciences. Of particular interest are catalysts which are both active and selective for the hydrogenation of CO_2_ to methanol. PdZn alloy nanoparticles supported on TiO_2_ via a solvent-free chemical vapour impregnation method are shown to be effective for this reaction. This synthesis technique is shown to minimise surface contaminants, which are detrimental to catalyst activity. The effect of reductive heat treatments on both structural properties of PdZn/TiO_2_ catalysts and rates of catalytic CO_2_ hydrogenation are investigated. PdZn nanoparticles formed upon reduction showed high stability towards particle sintering at high reduction temperature with average diameter of 3–6 nm to give 1710 mmol kg^−1^ h of methanol. Reductive treatment at high temperature results in the formation of ZnTiO_3_ as well as PdZn, and gives the highest methanol yield.

## Introduction

Atmospheric CO_2_ levels continue to increase, now inexorably linked with anthropogenic emissions and climate change [1]. The scientific community therefore faces a major challenge; to act collectively in curtailing global CO_2_ emissions whilst simultaneously exploring low-environmental impact routes to generating energy. Although usually considered a waste product, CO_2_ can be a sustainable carbon source for fuel synthesis. Provided that hydrogen is also generated from sustainable sources, the hydrogenation of CO_2_ to methanol could underpin the oft cited methanol economy, providing a non-fossil fuel derived energy storage, fuel and chemical feedstock [2]. Ideally, H_2_ would be produced via electrolysis of water utilising renewable energy. Whilst hydrogen is itself a good energy carrier, with high energy density per mass and clean combustion, it has low volume energy density. An integrated approach, with H_2_ generation and CO_2_ hydrogenation processes/plants in close physical proximity would be advantageous [3].

The utilisation of CO_2_ as a carbon source for methanol synthesis has been studied extensively, though is yet to be commercialised on a large scale. Methanol is therefore still produced on a world scale from synthesis gas (at 200–300 °C, 50–100 bar), which is itself the product of methane steam reforming (at *ca*. 850 °C, Ni-catalyst) [4, 5]. This two-step process incurs high energy and capital demands. Key barriers to CO_2_ hydrogenation replacing/supplementing this industry include catalyst stability and methanol yields. Another key consideration is reaction selectivity, as side reactions yield CO, CH_4_ and C_2_–C_4_ hydrocarbons which incur significant downstream separation costs. Additionally, methanol synthesis through direct CO_2_ hydrogenation with H_2_ is restricted by thermodynamic equilibria; hydrogenation to methanol is dominant at low reaction temperatures or high reaction pressure. Catalyst design is therefore vital, with a “good” catalyst for CO_2_ hydrogenation required to show strong adsorption and transportation of CO_2_, high concentration of hydrogenation sites, ability to stabilise intermediates and resistance towards water-induced deactivation. Cu has been extensively studied for CO_2_ hydrogenation, however it is often associated with deactivation on-stream [6]. This arises as catalytic activity is highly dependent on copper surface area whilst water, which is formed through the reverse water gas shift reaction, induces sintering of copper nanoparticles [7–9]. In contrast Pd, a well-known hydrogenation catalyst, is known to be stable towards water-induced sintering [10]. We previously reported Pd/ZnO to be an active catalyst for CO_2_ hydrogenation, with the choice of catalyst preparation method and Pd precursor shown to impact significantly upon product selectivity [11]. Investigation into the structural properties of Pd and PdZn species is key to understanding the origin of this catalytic activity, which will inform the designing of more methanol-selective catalysts. ZrO_2_-supported In_2_O_3_ showed high activity and 100% methanol selectivity, with this behaviour attributed to rapid formation and annihilation of oxygen vacancies during CO_2_ hydrogenation [12].

C=O dissociation has been identified as the rate determining step in CO_2_ hydrogenation, requiring 2.97 eV in the gas phase [13]. The catalytic reaction proceeds first through hydrogenation of CO_2_ to a formate intermediate [14], which then undergoes C–O bond dissociation and hydrogenation to form methoxide species [15]. Meanwhile, CO_2_ methanation requires two consecutive C–O bond dissociation events to yield carbon, which undergoes hydrogenation to form methane [13].

1 $${\text{C}}{{\text{O}}_2}+3{{\text{H}}_2} \to {\text{C}}{{\text{H}}_3}{\text{OH}}+{{\text{H}}_2}{\text{O }}\Delta {{\text{H}}_{298{\text{K}}}}= - 49.5\;{\text{kJ}}/{\text{mol}}$$

2 $${\text{C}}{{\text{O}}_2}+{{\text{H}}_2} \to {\text{CO}}+{{\text{H}}_2}{\text{O}}\,\Delta {{\text{H}}_{298{\text{K}}}}=+41.2{\text{ kJ}}/{\text{mol}}$$

3 $${\text{C}}{{\text{O}}_2}+4{{\text{H}}_2} \to {\text{C}}{{\text{H}}_4}+2{{\text{H}}_2}{\text{O}}\,\Delta {{\text{H}}_{298{\text{K}}}}= - 252.9{\text{ kJ}}/{\text{mol}}$$

In this study we prepared PdZn/TiO_2_ catalysts via solvent free chemical vapour impregnation (CVI) method to avoid surface contamination that can occur from the use of solvents. The influence of PdZn morphologies on the activity towards CO_2_ hydrogenation are reported. This will be achieved through studying the effect that reductive heat treatment conditions have upon physico-chemical properties of catalysts and their CH_4_, CO and CH_3_OH productivities.

## Experimental

All supported catalysts were prepared via CVI. The procedure for preparing 2 g of 5% PdZn/TiO_2_ (1Pd:5Zn molar ratio) is as follows; Pd(acac)_2_ (0.29 g, 0.939 mmol) and Zn(acac)_2_ (1.23 g, 4.698 mmol) were physically mixed with TiO_2_ (P25, 1.58 g, Sigma Aldrich) for 1 min. The dry mixture was transferred to a 50 ml Schlenk flask and then evacuated at room temperature (*ca*. 10^−3^ bar). Following this, the mixture was gradually heated to 145 °C. After 1 h the resulting pre-catalyst was recovered, prior to calcination in static air (500 °C, 16 h, 10 °C). All catalysts were reduced ex situ in a flow of 5% H_2_/Ar prior to assessment (at 400, 500 or 650 °C). For 7% PdZn/TiO_2_ and 10% PdZn/TiO_2_, a similar procedure was followed; increasing the wt% of Pd while a Pd: Zn molar ratio of 1:5. This method is referred as co-CVI.

Two sets of catalysts ^2^Pd–^1^Zn–TiO_2_ and ^2^Zn–^1^Pd–TiO_2,_ were also prepared using sequential CVI. For ^2^Pd–^1^Zn–TiO_2,_ Zn–TiO_2_ was first prepared as follows; Zn(acac)_2_ (1.23 g, 4.698 mmol) was physically mixed with TiO_2_ (P25, 1.58 g, Sigma Aldrich) for 1 min then was transferred to a 50 ml Schlenk flask. The mixture was then evacuated at room temperature (*ca*. 10^−3^ bar) and gradually heated to 145 °C. The system was then heated at this temperature for 1 h, after which it was allowed to cool to room temperature. Pd(acac)_2_ (0.29 g, 0.939 mmol) was then physically mixed with the Zn–TiO_2_ for 1 min. The resulting mixture was evacuated again at room temperature (*ca*. 10^−3^ bar) and gradually heated to 145 °C. After 1 h the resulting pre-catalyst was recovered, prior to calcination in static air (500 °C, 16 h, 10 °C). A similar procedure was repeated in preparing ^2^Zn–^1^Pd–TiO_2_, though Pd was added first, followed by Zn. All catalysts were reduced in situ at 400 °C in H_2_ (30 ml min^−1^, 1 h) unless stated otherwise.

Catalysts were characterised using a range of techniques. Powder X-ray diffraction (XRD) patterns were obtained at room temperature using an Enraf Nonus FR590 diffractometer fitted with a hemispherical analyser, using Cu Kα radiation (l 1/4 1.54 A). X-ray photoelectron spectra (XPS) were recorded on a Kratos Axis Ultra-DLD XPS spectrometer with a monochromatic Al Kα source (75–150 W) and analyser pass energies of 160 eV (for survey scans) or 40 eV (for detailed scans). Samples were mounted using a double-sided adhesive tape and binding energies referenced to the C (1s) binding energy of adventitious carbon contamination which was taken to be 284.7 eV. Data were analysed using Casa XPS software. To provide detailed morphological and compositional information at micro and nano-scales, samples were analysed on a JEOL 2100 (LaB6) high-resolution transmission electron microscope (HRTEM) system fitted with a high-resolution Gatan digital camera (2 k 2k) and a dark held HAADF/Z-contrast detector. Samples were suspended in DI water and *ca*. 1 μl was added to the TEM grid and dried. Lattice d-spacings were determined using Digital Micrograph software. Surface area analysis was performed on a Nova 2200e Quantachrome. The catalyst was pre-treated under vacuum at 250 C for 2 h before the surface area was determined by 5 point N_2_ adsorption at − 196 °C and the data was analysed using the BET method. Diffuse Reflectance Infrared Fourier Transform Spectroscopy (DRIFTS) spectra were collected on a Bruker Tensor 27 spectrometer fitted with a liquid N_2_-cooled MCT detector. Samples were housed in a Praying Mantis high temperature diffuse reflection environmental reaction chamber (HVC-DRP-4) fitted with zinc selenide windows.

Catalytic assessment for CO_2_ hydrogenation was carried out in a custom built fixed-bed continuous-flow reactor. The pelleted catalyst (0.5 g, 425–500 µm) was placed in a stainless tube reactor with internal diameter of 0.5 cm and length 50 cm, occupying a length of *ca*. 10 cm. Prior to testing, catalysts were pre-reduced in situ in a flow of H_2_ gas (30 ml min^−1^, 1 atm, 400 °C, 1 h) and subsequently cooled to room temperature. The system was then pressurised to 20 bar with the reactant gas (1 CO_2_: 3 H_2_: 1 N_2_ molar ratios), heated to 250 °C and reaction was carried out for 4 h. A standard reaction gas flow rate of 30 ml min^−1^ was used throughout (GHSV *ca*. 916 h^−1^). To avoid product condensation, post-reactor lines and valves were heated at 130 °C. Products were analysed via on-line gas chromatography using an Agilent 7890 system fitted with both FID and TCD detectors. Nitrogen was used as an internal standard. The reaction was carried out for 4 h.

## Results and Discussion

### Catalyst Characterisation

#### XRD Analysis

**Fig. 1:**
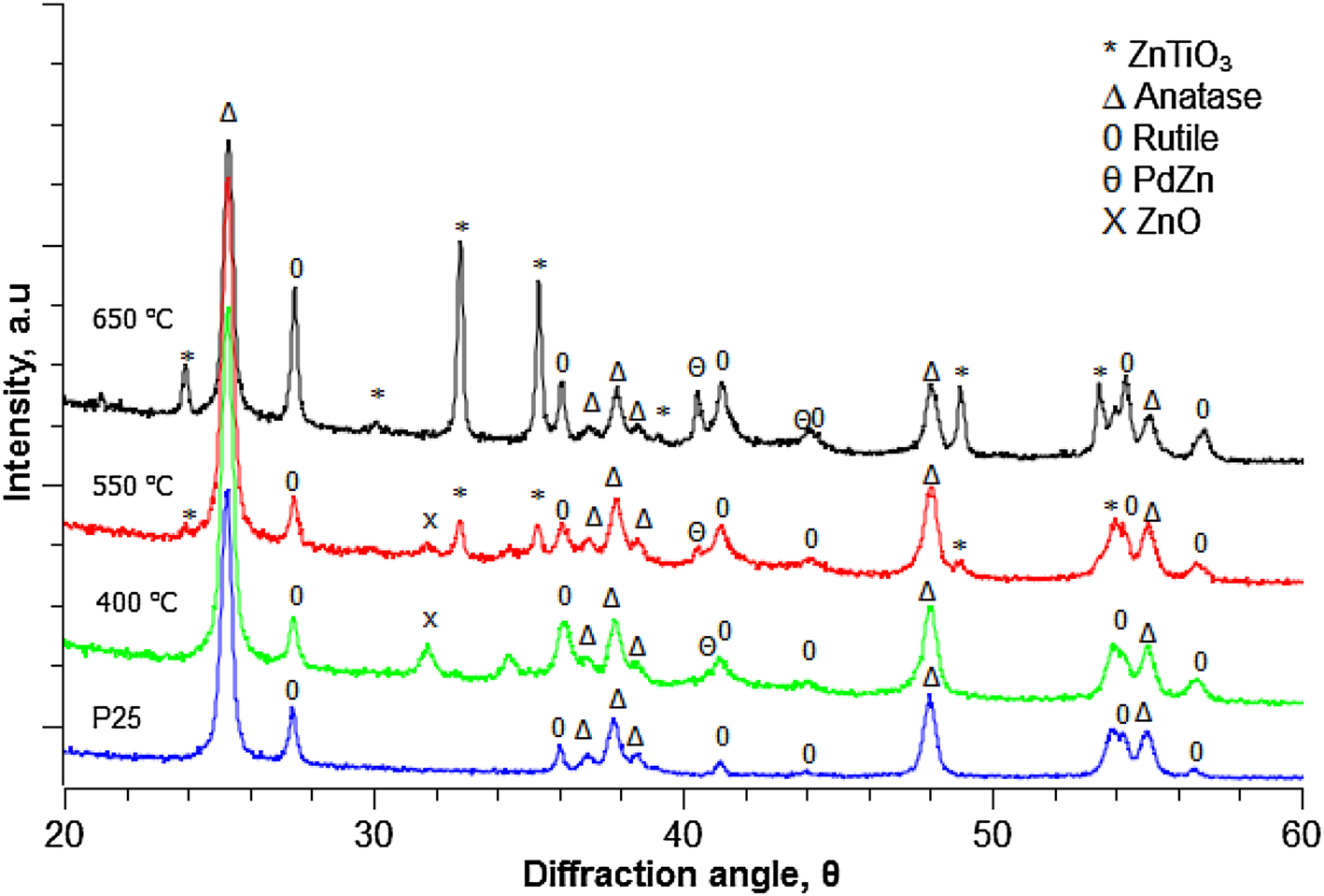
XRD of P25 and PdZn/TiO_2_ catalyst after hydrogen reduction at 400, 550 and 650 °C

XRD was used as a primary technique to confirm formation of the crystalline PdZn phase on TiO_2_. However, the (111) and (200) β-PdZn alloy reflections (at 41.2° and 44.1° respectively) [16] overlap with the (111) and (210) planes for rutile TiO_2_. This led to difficulty in detecting the PdZn alloy. Indeed, formation of this alloy can only be definitively identified in the XRD of the 5% PdZn/TiO_2_ which had been reduced for 1 h at 650 °C (Fig. 1). The broadening and increasing intensity of the reflections at 40.9° and 44.0° in comparison to the P25 TiO_2_ support implies formation of the PdZn alloy. Following reduction at 650 °C the PdZn alloy was clearly observed at 40.9° and 44.0°. Diffractions corresponding to a ZnTiO_3_ phase were also observed in Fig. 1 following reduction at 650 °C. Accompanied by a disappearance of the ZnO reflection at 31.6°, this suggests that incorporation of ZnO into the TiO_2_ lattice occurs during reduction at 650 °C.

To confirm whether high temperature reduction does indeed lead to a phase change, N_2_ physisorption studies were carried out. It is clear from Table 1 that the impregnation of Pd(acac)_2_ and Zn(acac)_2_ does not lead to a significant decrease in catalyst surface area, relative to unmodified TiO_2_ (P25) (Table 1, Entry 1). A gradual decrease in BET surface area is observed upon increasing the reduction temperature from 400 to 650 °C (Table 1, Entries 3 and 5). This can be attributed to TiO_2_ undergoing partial conversion from anatase to the rutile phase. Additionally, the formation of the ZnTiO_3_ phase also contributes to the reduction of the surface area [17]. From the XRD it can be seen that the ZnO phase disappears upon heating to 650 °C.

**Table 1:** The effect of reductive heat treatment conditions on the physico- chemical properties of 5% PdZn/TiO_2_

Entry	Reduction conditions^a^	PdZn size (nm)^b^	BET surface area (m^2^ g^−1^)^c^	B.E PdO (eV)^d^	B.E PdZn (eV)^d^	B.E Pd (eV)^d^
1	Unmodified TiO_2_	–	50	–	–	–
2	n/a	4.1^e^	43	336.6	–	–
3	5% H_2_/Ar, 400 °C, 1 h	3.9	44	–	335.8	335.0
4	5% H_2_/Ar, 550 °C, 1 h	4.4	39	–	336.1	335.0
5	5% H_2_/Ar, 650 °C, 1 h	5.6	38	–	336.1	335.0

^a^All catalysts were pre-calcined (500 °C, 10 °C min^− 1^, 16 h)

^b^Determined by TEM

^c^Determined by N_2_ physisorption

^d^Determined by XPS

^e^PdO particle size

**Fig. 2:**
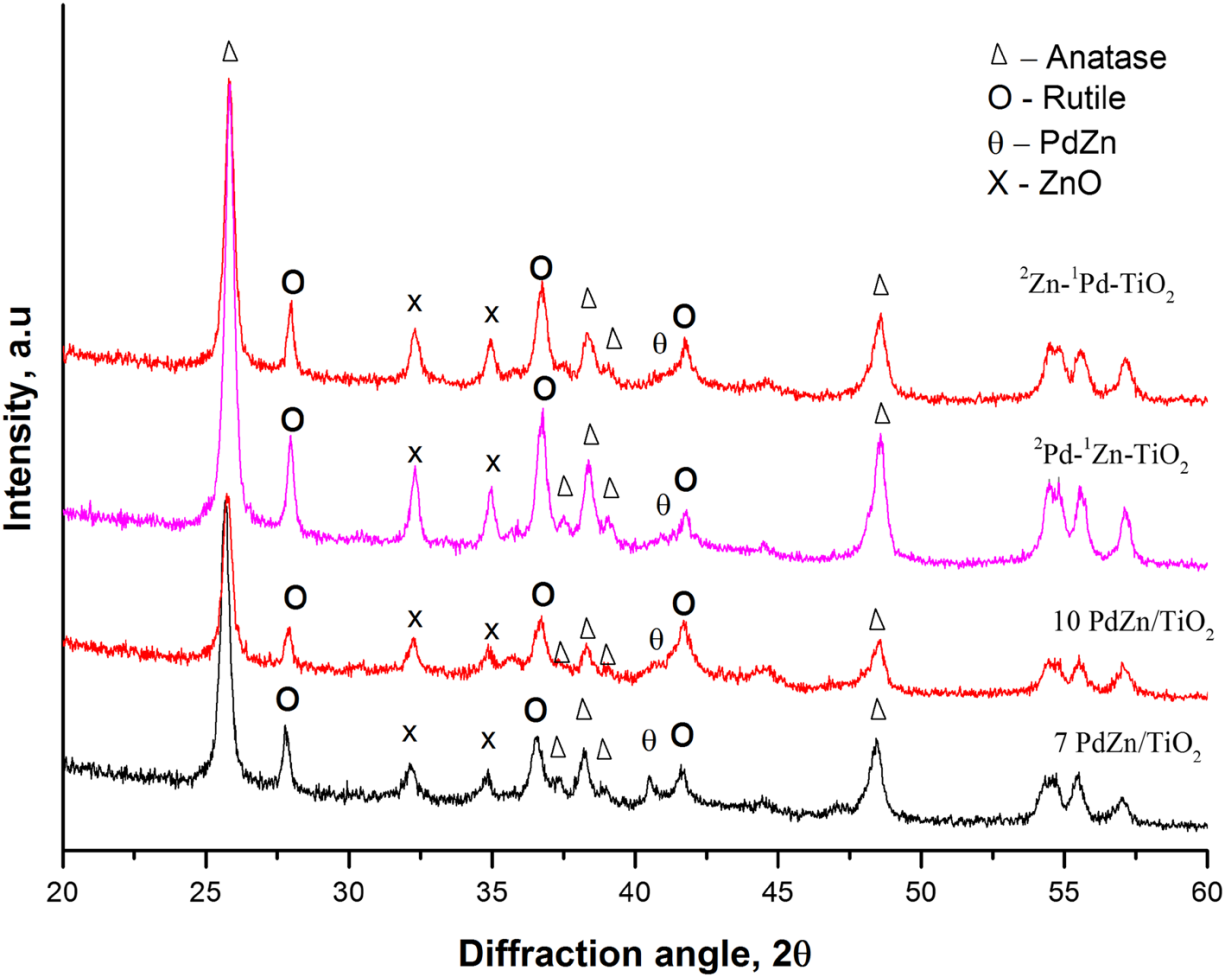
XRD analysis of 7 and 10 wt% PdZn/TiO_2_ catalysts; and catalysts prepared via sequential CVI, ^2^Pd–^1^Zn–TiO_2_ and ^2^Zn–^1^Pd–TiO_2_ after hydrogen reduction at 400 °C

X-ray diffractograms of 7% PdZn/TiO_2_ and 10% PdZn/TiO_2_ shown in Fig. 2. Whilst similar to that of 5% PdZn/TiO_2,_ the PdZn (111) peak at 40.78° is more pronounced for 7% PdZn/TiO_2_ than either 5 or 10% wt loading catalysts. Figure 2 also shows the diffraction patterns of the catalyst prepared via sequential CVI, ^2^Pd–^1^Zn–TiO_2_ and ^2^Zn–^1^Pd–TiO_2_. These samples showed relatively pronounced ZnO peaks (32.31° and 34.96°) when compared with 5% PdZn/TiO_2_ prepared by co-CVI method. ZnO crystallite size was determined using the Scherrer equation and ZnO peak at 32.31°. In this way ^2^Zn–^1^Pd–TiO_2_ showed a ZnO crystallite size of 40.6 nm and ^2^Pd–^1^Zn–TiO_2_ 31.3 nm. These values are significantly higher than the 29.0 nm determined for the analogous co-CVI catalyst.

#### XPS Analysis

**Fig. 3:**
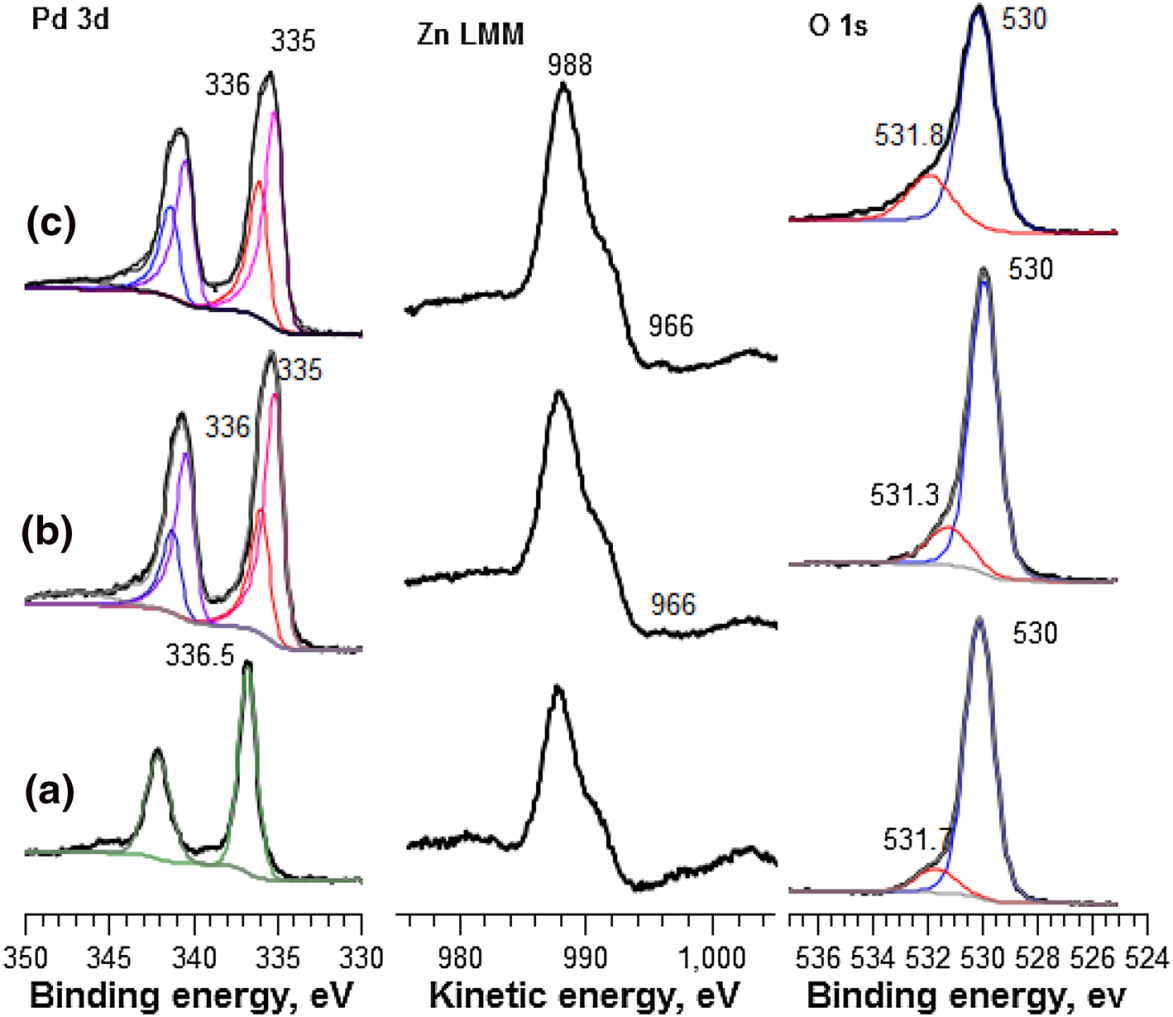
Pd 3d, Zn LMM Auger electron and O 1s XPS analysis of 5% PdZn/TiO_2_ following **a** calcination at 500 °C and subsequent reduction in H_2_ at **b** 400 °C and **c** 650 °C

The synthesised CVI catalysts were calcined in static air (500 °C, 16 h) to ensure complete removal of acetylacetonate precursors. XPS analysis of the calcined catalyst is shown in Fig. 3a. The Pd 3d, O 1s and Zn LMM Auger electron peak positions were calibrated using the C 1s signal at 284.8 eV. Transformation of Pd to the PdZn alloy can be observed in the Pd 3d signal. Following calcination at 500 (Fig. 3a), the peak is at 336.5 eV, which corresponds to oxidised Pd^2+^ in PdO. Following annealing in H_2_ (400 °C, 1 h) the peak shifts towards a lower binding energy of 335 eV and the appearance of a shoulder at 336 eV. The observed shift in binding energy occurs due to a change in the Pd environment, which depends on the localised charge on the emitting atom [18]. Indeed, a shift towards lower binding energies indicates a decrease in electronic charge, resulting from decreased electronegativity of adjacent substituents. This implies that the asymmetric peak at 335 eV corresponds to Pd^°^. Meanwhile, electronic changes occur in Pd due to the intermetallic bond with Zn upon forming PdZn, thereby effecting changes in the Pd localised charge. A correlation between the shoulder at 336 eV and formation of the PdZn alloy is apparent following reduction at 400 and 650 °C. PdZn alloy formation can be further established through close observation of the Zn LMM Auger electron region. The calcined catalyst shows a peak at K.E = 988 eV, which corresponds to ZnO. Meanwhile, a shoulder at 991 eV is often indicative of interstitial Zn on the catalyst surface. When reduced at 400 and 650 °C, a small peak appeared at 966 eV. This is similar to the Auger electronic position of Zn alloy [19, 20]. It is small because the oxide component is dominant formation of the Pd:Zn 1:1 alloy will only use 20% of the available Zn and also surface Zn metal may well oxidise on exposure to air.

Following calcination, the O 1s spectra showed two peaks at 530 and 531.7 eV. These represent two chemically distinct oxygen environments. The principal peak at 530 eV, is due to lattice oxygen and most likely originates from TiO_2_. The contribution at 531.7 eV can be attributed to chemisorbed oxygen, which can form through adsorption of water at surface oxygen vacancies [21, 22]. Reduction at 400 or 650 °C led the peak shoulder to increase in intensity. This is indicative of an increased concentration of surface defects, and suggests that these catalysts contain increased oxygen deficiency at the surface. XRD analysis confirms reaction of ZnO with TiO_2_ lattice to form a crystalline ZnTiO_3_ phase. Such a change has been reported to induce surface defects on TiO_2_ [23]. This is consistent with the appearance of chemisorbed oxygen at 531.7 eV, in the O 1s XPS spectrum [24].

**Fig. 4:**
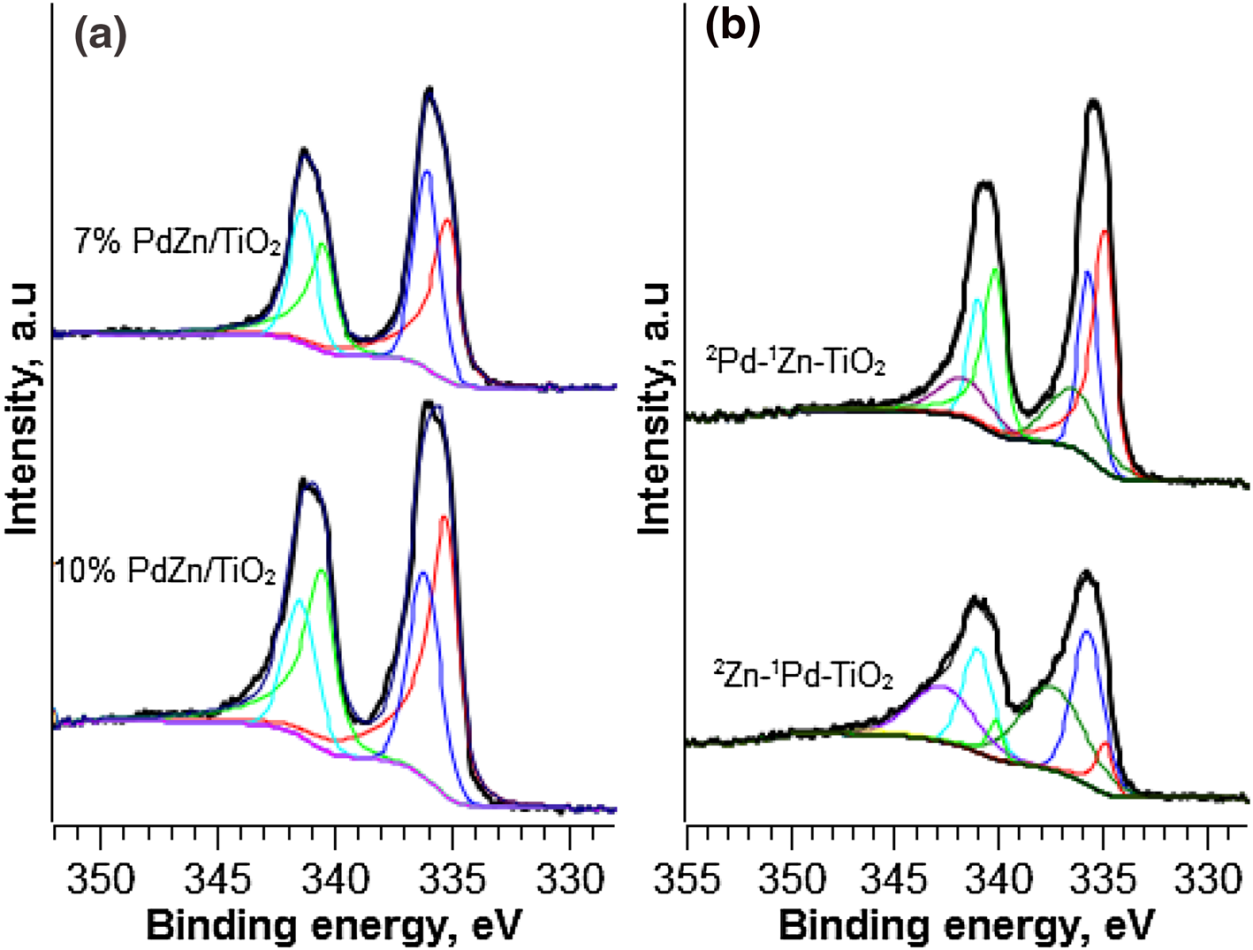
Pd 3d, XPS analysis of **a** 7% PdZn/TiO_2_ and 10% PdZn/TiO_2_ following reduction in H_2_ at 400 °C and **b** catalysts prepared via sequential CVI, ^2^Pd–^1^Zn–TiO_2_ and ^2^Zn–^1^Pd–TiO_2_ after hydrogen reduction at 400 °C

Figure 4a shows the Pd 3d regions in XPS spectra for PdZn/TiO_2_ at 7 and 10 wt% loadings. The catalysts were reduced prior to XPS analysis (H_2_, 400 °C, 1 h) to promote PdZn formation. Peaks corresponding to Pd^°^ (335 eV) and PdZn alloy (336 eV) were observed for both catalysts. However, based on the curve-fitted peak of Pd 3d signals, 7% PdZn/TiO_2_ showed a relatively high PdZn alloy: Pd° ratio. Figure 4b shows the Pd 3d region in XPS spectra for ^2^Pd–^1^Zn–TiO_2_ and ^2^Zn–^1^Pd–TiO_2_. In addition to PdZn (335 eV) and Pd° (336 eV) a peak at 337 eV, corresponding with PdO was observed.

#### CO Adsorption Studies

**Fig. 5:**
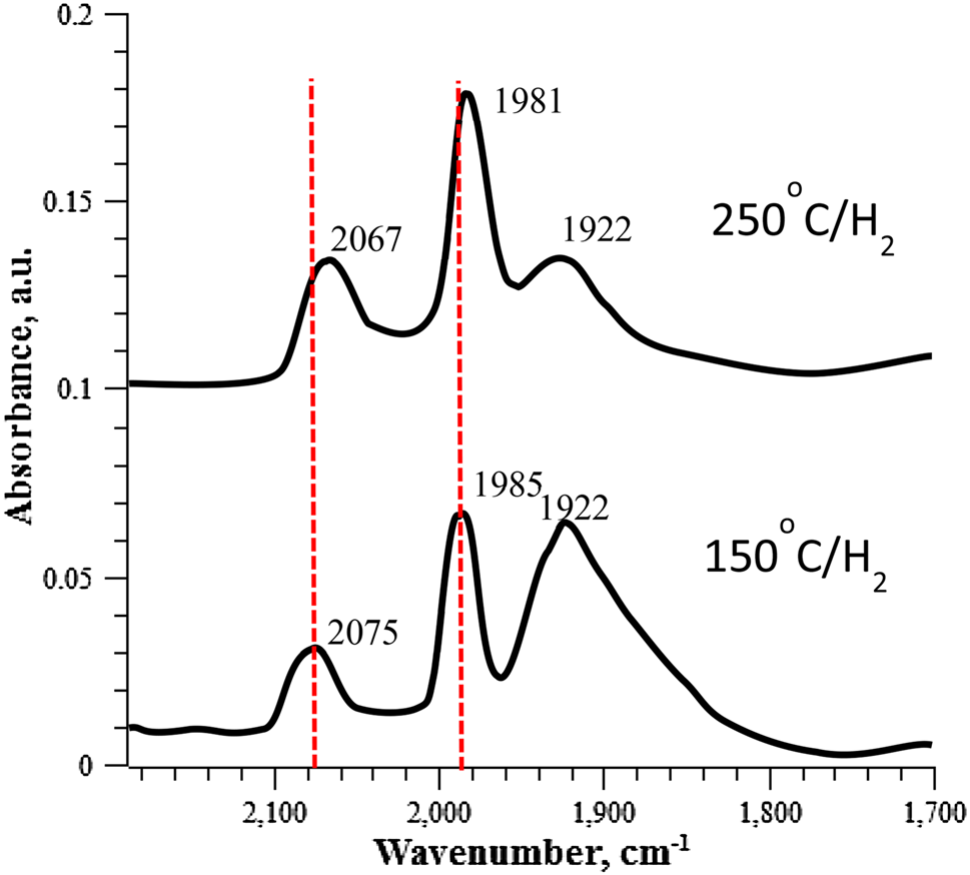
CO DRIFTS spectra of 5% PdZn/TiO_2_ following reduction at 150 and 250 °C

CO was used as a probe molecule to compare the surface adsorption properties of PdZn/TiO_2_ catalysts. Spectra are shown in Fig. 5. Following reduction at 150 °C (1 h), the DRIFTS spectrum of PdZn/TiO_2_ shows a band at 2075 cm^−1^, which corresponds to linearly adsorbed CO. Bands at 1985 and 1922 cm^−1^ are attributed to bridging CO species; at corner and edge sites respectively [25]. Bands associated with bridging CO are relatively intense when compared with the band a 2075 cm^−1^, suggesting that the catalyst surface consists mainly of metallic Pd. When reduced at 250 °C (1 h) a significant change in the CO feature is observed. In particular, the broad band at *ca*. 1922 cm^−1^, associated with bridging CO at edge sites, becomes less intense. These results suggest that reduction of the catalyst in hydrogen promotes formation of the PdZn alloy, as evidenced by disappearance of the bridging CO band at *ca*. 1922 cm^−1^. However, the surface is consists of Pd, which suggests the presence of isolated Pd species which require higher temperatures to alloy with Zn [26].

#### Surface and Morphological Analyses

**Fig. 6:**
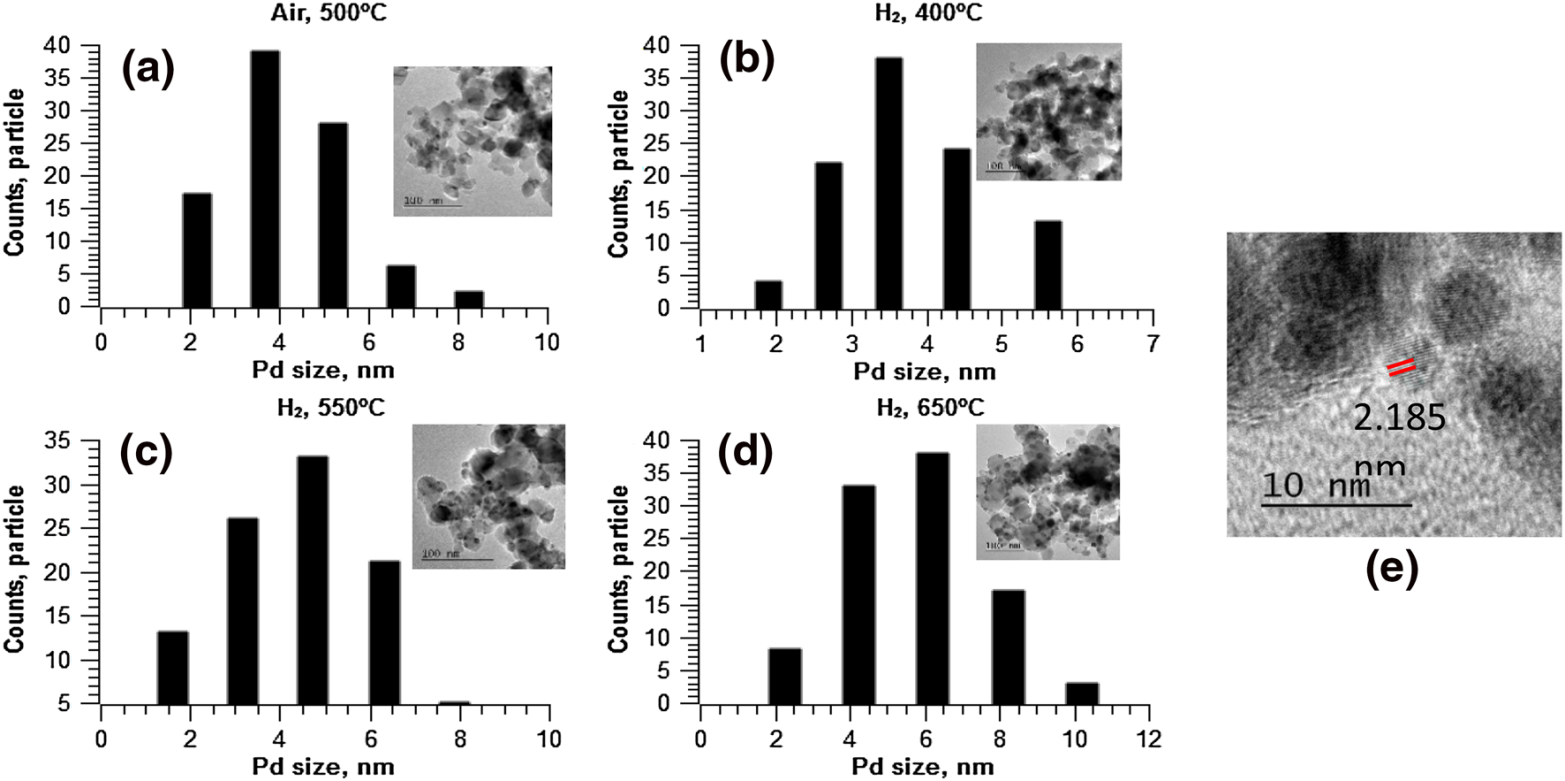
TEM images and particle size distribution histograms for 5% PdZn/TiO_2_ following; **a** calcination at 500 °C and subsequent reduction in H_2_ at **b** 400 °C, **c** 550 °C and **d** 650 °C. **e** A HR-TEM image of PdZn nanoparticles in 5% PdZn/TiO_2_ following reduction at 400 °C, showing a *d*-spacing calculation

**Fig. 7:**
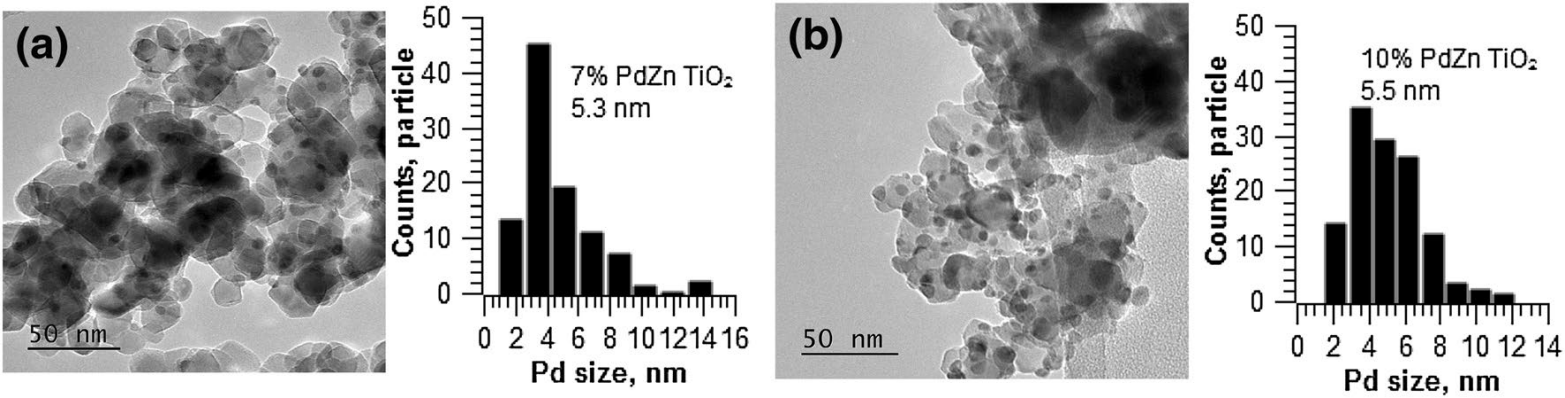
TEM images and particle size distribution of 7% PdZn/TiO_2_ and 10% PdZn/TiO_2_ after reduction at 400 °C

Morphological changes, ie the size and shape of nanoparticles within 5% PdZn/TiO_2_ following high temperature reductions (at 400, 550 and 650 °C) were studied via TEM analysis (Fig. 6). Representative micrographs and particle size distributions are shown in Fig. 3, with mean particle sizes summarised in Table 1. It is clear from Fig. 1a that CVI produced nanoparticles of *ca*. 4.1 nm average size, which are well dispersed on the TiO_2_ support. Reductive treatment at 400 °C led to a contraction in particle size (Fig. 3b, 3.9 nm). Further increase in reduction temperature, to 550 or 650 °C, led to particle growth (4.4 and 5.6 nm respectively). High resolution TEM analysis following reduction at 400 °C, reveals a d-spacing of 0.21 nm on some metal particles, which corresponds to the PdZn (111) lattice plane of PdZn nanoparticles [27, 28]. TEM analysis of 7 and 10 wt% PdZn/TiO_2_ shows that CVI affords evenly distributed PdZn nanoparticles across the surface of TiO_2_ (Fig. 7). Furthermore, particle size analysis shows the mean particle size to increase with metal loading, from 5.3 nm (± 2.3 nm) at 7 wt% PdZn to 5.5 nm (± 1.9 nm) for 10 wt% PdZn on TiO_2_.

### Catalytic Activity

Characterisation studies showed a general increase in PdZn nanoparticle size upon increasing reductive heat treatment temperature from 400 to 650 °C. A corresponding decrease in BET surface area was attributed to an increasing degree of transformation to rutile TiO_2_ and ZnTiO_3_ phases. To determine whether CO_2_ hydrogenation on PdZn is structure-sensitive, these catalysts were assessed under standard reaction conditions (P = 20 bar, 1 CO_2_: 3 H_2_: 1 N_2_, 30 ml min^−1^, 250 °C). The results in Table 2 show no defined correlation between reduction temperature and CO_2_ conversion. Indeed, the samples reduced at 400, 550 and 650 °C afforded 10.1, 10.5 and 9.1% conversion respectively (Table 2, Entries 1 and 3). Across this range however, an appreciable increase in methanol productivity was observed, reaching 1710 mmol kg_cat_^−1^ h^−1^ (58.6% selectivity, 5.3% yield) when the catalyst was treated at 650 °C. Another key observation is the relationship between reduction temperature and CO_2_ methanation rates, with CH_4_ productivity being immeasurable when the catalyst was reduced at 650 °C. In industrial methanol synthesis processes, CH_4_ formation incurs significant downstream separation costs. Metallic Pd is a well-known methanation catalyst and this is likely to occur at the Pd-metal oxide interface [13, 29]. It is clear from data in Table 2 that PdZn alloy formation significantly inhibits methane formation, thereby suggesting a high degree of PdZn alloying. When reduced at 400 °C, the catalyst has some limited activity for CO_2_ methanation, with a CH_4_ productivity of 8 mmol kg_cat_^−1^ h^−1^ (*ca*. 0.1% selectivity). This suggests the presence of non-alloyed Pd species, for example isolated Pd°. For commercial applications, even 0.1% CH_4_ selectivity would prove problematic and expensive [30, 31]. Complete suppression of CH_4_ formation can be achieved through reduction at 650 °C as shown in Table 2 Entry 3 (note; the GC-FID detection limit for CH_4_ was 1 ppm or ~ 0.0005% effective yield). Studies summarised in Tables 1 and 2 do not show a definitive correlation between reduction temperature, physico-chemical properties (BET surface area, PdZn particle size) and catalyst performance. However, XPS analysis did show a shift in the position of the PdZn peak towards higher binding energies with increasing reduction temperature, suggesting a high degree of alloying with Zn. Meanwhile XRD analysis showed incorporation of ZnO into the TiO_2_ following reduction at 650 °C, leading to formation of a ZnTiO_3_ phase. Given the relatively high methanol productivity shown by PdZn/TiO_2_ following reduction at 650 °C, we therefore suggest that the presence of ZnTiO_3_ promotes CO_2_ hydrogenation through increasing the number of available surface oxygen vacancies.

**Table 2:** The effect of reduction temperature on catalytic activity

Entry	Reduction temperature (°C)	χ CO_2_ (%)	S (CH_3_OH) (%)	S (CO) (%)	S (CH_4_) (%)	mmol (CH_3_OH) (kg_cat_^−1^ h^−1^)	mmol (CO) (kg_cat_^−1^ h^−1^)	mmol (CH_4_) (kg_cat_^−1^ h^−1^)
1	400	10.1	40	59	0.1	1420	1550	8
2	550	10.5	35	64	0.05	1190	2170	3
3	650	9.1	58	41	0	1710	1210	0

Catalytic data at 4 h of reaction

All catalysts were pre-calcined (500 °C, 10 °C min^−1^, 16 h)

To further study whether the structure of PdZn nanoparticles has a significant effect upon catalyst activity, 5% PdZn/TiO_2_ was prepared by CVI with Pd and Zn impregnated onto TiO_2_ in either one (co-CVI) or two (sequential) steps. The co-CVI catalyst was prepared as detailed in the experimental. Meanwhile for sequential CVI catalysts monometallic Pd/TiO_2_ and Zn/TiO_2_ were first prepared, followed by impregnation of the second metal, calcination in static air (500 °C, 16 h) and in situ reduction (400 °C, 1 h, 30 ml min^−1^ H_2_).The performance of these catalysts is summarised in Table 3. CO_2_ conversion showed a strong dependence on the preparation method. Co-impregnation of Pd and Zn (Table 3, Entry 3) led to a higher CO_2_ conversion and methanol productivity than when metals were impregnated sequentially (Table 3, Entries 1 and 2).

**Table 3:** Catalytic activity of 5% PdZn/TiO_2_ catalysts prepared by CVI with metals impregnated either sequentially or simultaneously

Entry	Catalyst^a^	χ CO_2_ (%)	S (CH_3_OH) (%)	S (CO) (%)	S (CH_4_) (%)	mmol (CH_3_OH) kg_cat_^−1^ h^−1^	mmol (CO) (kg_cat_^−1^ h^−1^)	mmol (CH_4_) (kg_cat_^−1^ h^−1^)
1	^2^Pd–^1^Zn–TiO_2_	8.6	39	60	0.2	1070	1670	5.8
2	^2^Zn–^1^Pd–TiO_2_	6.7	11	88	0.2	247	1910	4.5
3	PdZn/TiO_2_	10.1	40	59	0.1	1420	1550	8

^a^All catalysts were pre- calcined (500 °C, 10 °C min^−1^, 16 h) and reduced in situ (400 °C, H_2_, 1 h) prior to reaction, ^1^ and ^2^ denote the order of sequential metal impregnations

Sequential addition of Pd to Zn/TiO_2_ (^2^Pd–^1^Zn–TiO_2_) affords 8.6% CO_2_ conversion, whilst the inverse ^2^Zn–^1^Pd–TiO_2_ catalyst shows notably lower CO_2_ conversion of 6.7%. XPS analysis (Fig. 4b) showed the presence of PdO on the surface and XRD (Fig. 2) revealed larger ZnO crystallites for catalysts prepared via sequential CVI. This suggests that co-addition of Pd and Zn affords a higher degree of Pd–Zn alloying. It is difficult to assign formation of the PdZn alloy to specific differences between these synthesis methods (Table 3, Entry 3), but is likely that there is an interaction between Pd(acac)_2_ and Zn(acac)_2_ precursors. This might promote formation of the PdZn active site. Unfortunately, due to the instability of acetylacetonate precursors, it was not possible to study such interactions spectroscopically.

Another approach to increase methanol yields would be to increase the concentration of active sites on the TiO_2_ support. PdZn alloy nanoparticles have previously been reported as both the active site and a stabiliser of formate intermediates [11, 32]. With an aim to increase the number of PdZn sites the Pd loading was increased to 7 and 10 wt%. Across this range, the Pd: Zn molar ratio was maintained at 1:5 as this was previously reported to afford optimal methanol yields [32]. Data in Table 4 summarises the performance of these catalysts; CO_2_ conversion and product selectivities, at temperatures of between 190 and 250 °C.

**Table 4:** The catalytic activity of 5 wt% PdZn/TiO_2_, 7 wt% PdZn/TiO_2_ and 10 wt% PdZn/TiO_2_ (Pd: Zn = 1: 5 molar) at different reaction temperatures

Entry	Catalyst^a^	T (°C)^b^	χCO_2_ (%)	S (CH_3_OH) (%)	S (CO) (%)	mmol (CH_3_OH) (kg_cat_^−1^ h^−1^)	mmol (CO) (kg_cat_^−1^ h^−1^)
1	5% PdZn(1:5)/TiO_2_	190	1.5	97	2	592	17.8
		210	2.5	59	40	606	411
		230	5.7	46	53	1090	1250
		250	10.1	40	59	1420	1550
2	7% PdZn(1:5)/TiO_2_	190	3.5	99	1.0	1110	11
		210	5.3	90	9	1560	162
		230	7.1	79	20	1810	473
		250	10.3	61	38	2040	1280
3	10% PdZn(1:5)/TiO_2_	190	3.8	66	33	823	423
		210	4.2	78	22	855	252
		230	4.9	60	39	961	630
		250	5.7	23	77	426	1430

^a^All catalysts were pre- calcined (500 °C, 10 °C min^− 1^, 16 h) and followed by reduction (400 °C, H_2_, 1 h)

^b^Reaction temperature. Catalytic data at 4 h of reaction

7 wt% PdZn/TiO_2_ and 5 wt% PdZn/TiO_2_ showed comparable CO_2_ conversion at a reaction temperature of 250 °C (*ca*. 10%). Further increasing the Pd loading to 10 wt% resulted in a decrease in catalytic activity at high temperatures, with CO_2_ conversion of 5.7%. It is likely that the maximum loading of well-dispersed metal, achievable in CVI, is restricted such that high dispersion is dependent on close proximity between acetylacetonate precursor and support. TEM analysis shows the mean PdZn particle size to increase with increasing loading. It likely that the low activity observed for the 10 wt% PdZn catalyst stems from poor distribution of the acetylacetonates across the support surface. Furthermore, at such high loadings, it is possible that calcination for 16 h (at 500 °C) was not sufficient to remove all carbon species. Residual carbon contaminants on the catalyst surface would be expected to have a detrimental effect upon catalytic activity [32].

The effect of varying reaction temperature on the performance of these catalysts is shown in Table 4. A general trend towards higher conversion was observed with increasing reaction temperature. For example, conversion over 5% PdZn/TiO_2_ increased from 1.5 to 10.1% moving from 190 to 250 °C. This was associated with increased methanol productivities, from 592 mmol kg_cat_^−1^ h^−1^ to 1420 mmol kg_cat_^−1^ h^−1^. However, high temperatures do not make CO_2_ hydrogenation more kinetically favourable, as CO_2_ methanation and reverse water gas shift reactions become more favourable and consequently affect methanol selectivity. It is interesting that although 5 and 7 wt% catalysts comparable CO_2_ conversion at 250 °C, activities at lower reaction temperatures differ greatly. At 190 °C, the lowest reaction temperature at which the 7 wt% catalyst was able to initiate CO_2_ conversion, 3.5% CO_2_ conversion and 97% methanol selectivity were observed (1110 mmol (CH_3_OH) kg_cat_^−1^ h^−1^). The 7 wt% catalyst also showed superior activity to 5 wt% at both 210 and 230 °C. We consider this to be an important observation as it suggests that the catalytic hydrogenation of CO_2_ to methanol is likely to be restricted by thermodynamic equilibria at higher reaction temperatures. However, at temperatures of below 250 °C, where the reaction is kinetically as opposed to thermodynamically limited, an increase in the concentration of active sites significantly improves methanol productivity.

## Conclusions

CVI is an effective method for preparing highly dispersed, alloyed PdZn nanoparticles with mean diameters in the region of 3–6 nm. Reductive heat treatment of these catalysts leads to improved methanol productivities and has been shown to significantly inhibit undesirable methane formation reactions. Spectroscopic studies show that when reduced at a temperature of 650 °C, a ZnTiO_3_ phase forms, which correlates with an increase in methanol yield. Preparation of PdZn/TiO_2_ catalysts whereby Pd and Zn were added sequentially, highlights the importance of interaction between Pd(acac)_2_ and Zn(acac)_2_ in forming the PdZn alloy active site, with inactive PdO and ZnO phases forming during these preparations. These catalysts were, in turn, less active than co-CVI analogues, further showing the role of the PdZn alloy in catalysing CO_2_ hydrogenation. Future studies will probe the role that metal acetylacetone-metal acetylacetonate interactions play in formation of the desired PdZn alloy.
